# New Advances in Viral and Microorganism Disinfectants

**DOI:** 10.3390/microorganisms11102530

**Published:** 2023-10-10

**Authors:** Takashi Onodera, Rikio Kirisawa

**Affiliations:** 1 Research Center for Food Safety, Graduate School of Agricultural and Life Sciences, University of Tokyo, Bunkyo-ku, Tokyo 113-8657, Japan; 2 School of Agriculture and Life Science, University of Tokyo, Bunkyo-ku, Tokyo 113-8657, Japan; ar-kirisa@g.ecc.u-tokyo.ac.jp

Nanomaterials are used to develop simpler, cheaper, and faster methods for disease diagnosis [1]. Recently, owning to the coronavirus COVID-19 pandemic, intervention in viral transmission has become an important priority in public health. Disinfectants, such as weakly acidic hypochlorous acid solution and ethanol, are known to destroy lipids in the viral envelope of the severe acute respiratory syndrome coronavirus SARS-CoV-2. However, ethanol is flammable and can cause harmful injury to human skin. Chemical reactions with ethanol cause the structural decay of chemical synthetic fibers, plastic, and cotton clothes. Sodium hypochlorous acid solution (pH > 10) has a weaker virucidal effect, when used at concentrations of <200 ppm, and a more concentrated solution is required to achieve efficient effects [2].

A weakly acidic hypochlorous acid solution (pH 6) is 10–50-fold more efficient against microorganisms [2]. However, when a weakly acidic hypochlorous acid solution is used at a concentration of <50 ppm, the microbiocidal effects are easily lost in the presence of organic matter contamination. Temperatures of higher than >25 °C and ultraviolet (UV) rays from sunshine influence the microbiocidal effects of this disinfectant [3]. When a weakly acidic hypochlorous acid solution is used at concentration of >100 ppm, chloramine and chloride gases are produced. These gases exhibit corrosive effects on steel. Therefore, environmentally friendly and non-harmful disinfectants for viruses and microorganisms are still under development. Numerous types of virucidal measures, such as cold gas plasma [4], chemical disinfectants [5], 222 nm UV [6], and photocatalysis [6], have been actively studied and are reported in this collection.

This Special Issue consists of one review, four original articles, and two communication articles. These discuss various new photocatalysts, naturally produced disinfectants (Kampo), and nanomaterials that function as disinfectants. Nanomaterials can prevent viral contamination via air and through contact with contaminated surfaces and can sterilize protective equipment, especially in hospital settings and animal facilities [6,7]. Creating a self-disinfecting surface using graphene–silver hybrids is a promising strategy to prevent the spread of COVID-19 [8].

Several products made of nanoparticles exhibit antimicrobial activities and are used for the disinfection of surfaces. CAC-717 is a novel disinfectant consisting of calcium bicarbonate mesoscopic crystals that contain mesostructured nanoparticles, which inactivate enveloping and non-enveloping viruses and prion [9,10]. Graphene in face masks can also sterilize SARS-CoV-2 and allow reuse [11]. Therefore, this coating is suitable for use on surfaces in public places.

Ephedrin alkaloids (EFE) and herbal extracts exert anti-viral effects against the influenza virus [12,13]. The unpredictable and unknown nature of COVID-19, the similarity of the specific properties of this disease, and the physicochemical properties of nanosystems will lead to a solution based on new technologies [14,15,16]. Kampo medicine is a traditional Japanese medicine consisting of natural herbs and animal products. In Kampo medicine, several compounds have been reported to have anti-viral effects. Recently, these extracts were shown to exhibit anti-viral effects against human coronaviruses and SARS-CoV-2 [15,16]. EFE has been widely studied by virologists at the National Institute of Health Science (NIHS), in Japan [16]. At the NIHS Biosafety Level (BSL)-3 laboratory, Vero E6/TMPRSS2 cells were cultivated and infected with SRAS-CoV-2 (moi = 0.03). After 2 h, the cells were washed with media and cultured for an additional 3 h. Real-time polymerase chain reaction (PCR) was used to determine the number of N protein genome (SARS-CoV-2) copies inside the cells. EFE (5–100 μg/mL) and ephedra herb macromolecule condensed tannin (EMCT; 5 μg/mL) was added to the cells 1 h before the infection [16]. The same concentration of the anti-viral agent was added during cultivation. The cytotoxic effect of the anti-viral agent was tested using the 3-(4,5-di-methylthiazol-2-yl)-2,5-diphenyltetrazolium bromide (MTT) assay [16].

In the EFE-treated groups, the increase in viral RNA was suppressed in a dose-dependent manner. EFE suppresses viral growth in the early phases of viral replication during the cell entry process of virion attachment to the viral receptor [16]. EMCT showed similar effects. At the indicated doses, the MTT assay showed that EFE and EMCT did not exhibit any cytotoxicity. Therefore, EFE and EMCT may be useful for the early treatment of COVID-19.

Another Kampo product has been studied by scientists at the University of Tokyo [15]. Epigallocatechin gallate (EGCg) is catechin derived from green tea that is traditionally used as a virucidation product. EGCg was enclosed inside cyclodextrin molecules (CD-EGCg) molecules to enhance their solubility in water. Higher concentrations of EGCg in water enhance virucidal effects against numerous types of viruses [15]. Human influenza viruses and coronaviruses are widely known to be affected by EGcg. However, CD-EGCg did not show any cytotoxicity during the cultivation of the MDBK cell line without a viral infection. CD-EGCg exhibited a virucidal effect after the adsorption of viruses onto the cell line. However, CD-EGCg did not show virucidal effects against free viruses in media. It was suggested that CD-EGCg could be used to spray raw food at cold temperatures [15].

Various antibodies to cytokines against cytokines has been shown to inhibit disease progression and virus-induced immunopathology [17,18,19,20,21]. Most patients with severe COVID-19 show increased levels of serum pro-inflammatory cytokines such as interleukin (IL)-6, IL-1β, IL-2, IL-8, and IL-17. In lethal COVID-19 cases, SARS-CoV-2 nucleocapsid antigen has been detected in a sizable portion of adipocytes [22]. This shows that the virus may directly infect the parenchymal cells of subcutaneous fat. The infection appeared to activate the interferon pathway and attract infiltrating leukocytes. The adipose tissue serves as a significant reservoir of SARS-CoV-2 and is an important source of mediators causing immunopathology [22]. In addition, the reactivation of latent cytomegalovirus and Epstein–Barr virus (EBV) infection may occur in COVID-19 patients, causing fatigue or neurological symptoms such as brain fog [23]. In this sense, new advances in the disinfectants of viruses and antiviral agents are crucial. Collectively, these new drugs can inhibit the first steps of virus-induced immunopathology.
